# Enhanced microstructure and mechanical properties of ZrN-reinforced AlSi10Mg aluminum matrix composite

**DOI:** 10.1038/s41598-024-58614-6

**Published:** 2024-05-02

**Authors:** Veronika Suvorova, Sergey Volodko, Dmitrii Suvorov, Stanislav Chernyshikhin, Andrey Nepapushev, Artem Korol, Lidiya Volkova, Pavel Sokolov, Alexander Khort, Dmitry Moskovskikh

**Affiliations:** 1 University of Science and Technology MISIS, Moscow, Russia; 2 Institute of Nanotechnology of Microelectronics of the Russian Academy of Sciences, Moscow, Russia; 3 KTH Royal Institute of Technology, Stockholm, Sweden

**Keywords:** Metal-matrix composites, Powder metallurgy, High-energy ball milling, Spark-plasma sintering, Mechanical properties, Materials science, Materials chemistry

## Abstract

Aluminum matrix composites (AMCs), incorporating Zirconium Nitride (ZrN) as reinforcing additives, demonstrate immense promise for applications in aerospace, automotive, and power generation due to their unique combination of low density, superior mechanical properties, and excellent thermal/electrical conductivity. This study explores the influence of ZrN reinforcement on the microstructure and mechanical properties of AlSi10Mg metal-matrix composites. Utilizing high-energy ball milling (HEBM) and spark-plasma sintering (SPS), ZrN/AlSi10Mg composites were synthesized, achieving nearly full density with uniform ZrN distribution, while phase and chemical transformations were not observed in the bulk composites. The addition of ZrN resulted in a notable increase in hardness of 237% (182 ± 8 HV_2_), elastic modulus of 56% (114 ± 3 GPa), compressive and tensile strength of 183% (565 ± 15 GPa), and 125% (387 ± 9 GPa), respectively, for composites containing 30% ZrN, compared to the non-reinforced alloy. Experimentally determined coefficients of thermal expansion (CTEs) for composites with 10%, 20%, and 30% ZrN content were 19.8 × 10^−6^ °C^−1^, 19.1 × 10^−6^ °C^−1^, and 18 × 10^−6^ °C^−1^, respectively, which well relates to Schapery’s model. These findings contribute to understanding the synthesis, mechanical behavior, and thermal properties of ZrN/AlSi10Mg composites, demonstrating their potential for diverse engineering applications.

## Introduction

Lately, among aluminum alloys, great attention has been paid to aluminum–magnesium–silicon (Al–Si–Mg) alloys, particularly to AlSi10Mg, owing to their high corrosion resistance, good workability and weldability, and high thermal conductivity^1–3^. This makes Al–Si–Mg alloys prospective for use in the aerospace and marine industry. However, in comparison with other aluminum alloys, AlSi10Mg has low hardness, wear resistance, and tensile strength. In addition, these alloys have a high coefficient of thermal expansion, which also limits their use. In this regard, improving the performance characteristics of AlSi10Mg is an important task.

Frequently, such ceramic compounds like SiC^4,5^, Al_2_O_3_^6^, B_4_C^7^, TiC^8^, Si_3_N_4_^9^, TiB_2_^10^, BN^11^, ZrC^12^, etc. serve as reinforcing additives to aluminum alloys, as well as various forms of carbon^13,14^ and intermetallics^15–17^. Zirconium nitride is an equally interesting additive for improving the mechanical and functional properties, for example, radiation resistance, of metals and alloys. ZrN has good chemical stability, high hardness (20 GPa)^18^, corrosion and radiation resistance^19,20^, low resistivity^21^, and friction coefficient^22^, as well as coefficient of thermal expansion (CTE) (7.24 × 10^−6^ К^−1^)^23^. For example, the compressive strength of ZrN/W composites increases with increasing ZrN content and reaches a maximum at 50 vol% ZrN, whereas the addition of 10 vol% ZrN to the W matrix increases the flexural strength almost twice^24^. Musil et al.^25^ prepared composite nc-ZrN/Cu coatings by magnetron sputtering, which demonstrated ultra-high hardness (55 GPa). While ZrN particles have demonstrated superior effects on the functional properties of various composites and exhibit high potential for enhancing the characteristics of aluminum-based composites, their impact on the mechanical and thermal properties of aluminum alloys remains unexplored. This knowledge gap sparks interest in the investigation of AMCs reinforced with ZrN.

Various methods, including conventional approaches, such as gas pressure infiltration, friction stir processing, stir/squeeze casting, and equal channel angular pressing (ECAP)^26^ have been employed to fabricate AMCs. However, the conventional methods exhibit drawbacks such as the non-uniform distribution of reinforcing agents in a matrix, attributed to differences in densities and poor wettability. In contrast, powder metallurgy methods emerge as the most promising for producing AMCs. Previous studies have demonstrated that ball milling can achieve a uniform distribution of reinforcing particles in a metallic matrix^27^. On the other hand, for the production of bulk materials with low porosity and high mechanical properties, the spark plasma sintering method appears particularly suitable owing to its high heating rate, rapid compaction, and inhibiting grain growth^12,28,29^. For instance, Ji et al.^2^ prepared SiC/AlSi10Mg composites by planetary ball milling, spark plasma sintering (SPS), and hot extrusion composite preparation process. This approach enabled to obtaining of high-density materials with an elongation of 11.7% and tensile strength up to 197 MPa, which is 21% higher than that for AlSi10Mg. Moreover, the usage of powder metallurgy allowed for preventing undesired chemical reactions in the phase boundary owing to low sintering temperature. Another example of the effectiveness of the spark plasma sintering method for producing composite materials is the work of Sabahi Namini et al.^30^. Ti–4.8% wt. TiB_2_ composites sintered under optimal parameters demonstrated a high relative density of 99.88% along with high compressive ultimate strength (541 MPa), elongation (6.62%), and macro (428 HV30) and microhardness (501 HV0.3).

Herein, we study the effect of ZrN reinforcement on the microstructure, mechanical properties, and CTE of the lightweight and corrosion-resistant aluminum alloy AlSi10Mg metal-matrix composites. The composite was obtained using high-energy ball milling (HEBM) with subsequent SPS of the powders. The applied synthesis approach resulted in the formation of a nearly fully dense ZrN/AlSi10Mg composite with a uniform distribution of ZrN particles within the AlSi10Mg matrix. It was shown that the addition of ZrN significantly enhanced the mechanical properties of the AlSi10Mg composites, leading to substantial increases in hardness, elastic modulus, compressive, and tensile strength. The effect can be related to both strain hardening during the HEBM process and the reinforcement effect by the ZrN grains. The addition of ZrN has been shown to reduce CTE. Theoretical calculations shown in the study enable the assessment of a desired amount of ZrN for the usage of the composites in electronic devices.

## Materials and methods

### Feedstock powders

AlSi10Mg powder of spherical morphology (Fig. S1a) with an average particle size of ~ 42 μm (Fig. S1b) was used as a matrix material, and its chemical composition is given in Table S1. The reinforcing ceramic component was ZrN obtained by combustion synthesis^31,32^. ZrN particles have a fragmented morphology (Fig. S1c) with an average size of ~ 2 μm (Fig. S1d). The diffraction patterns of the initial powders are shown in Fig. S2. As can be seen, AlSi10Mg consists of two phases: α-Al Fm-3m (225) and Si Fd-3m (227). Meanwhile, the diffraction pattern of ZrN contains peaks corresponding only to a cubic phase Fm-3m (225), which indicates that the synthesized powder is single-phase.

### Fabrication of bulk ZrN/AlSi10Mg composites

**Figure 1:**
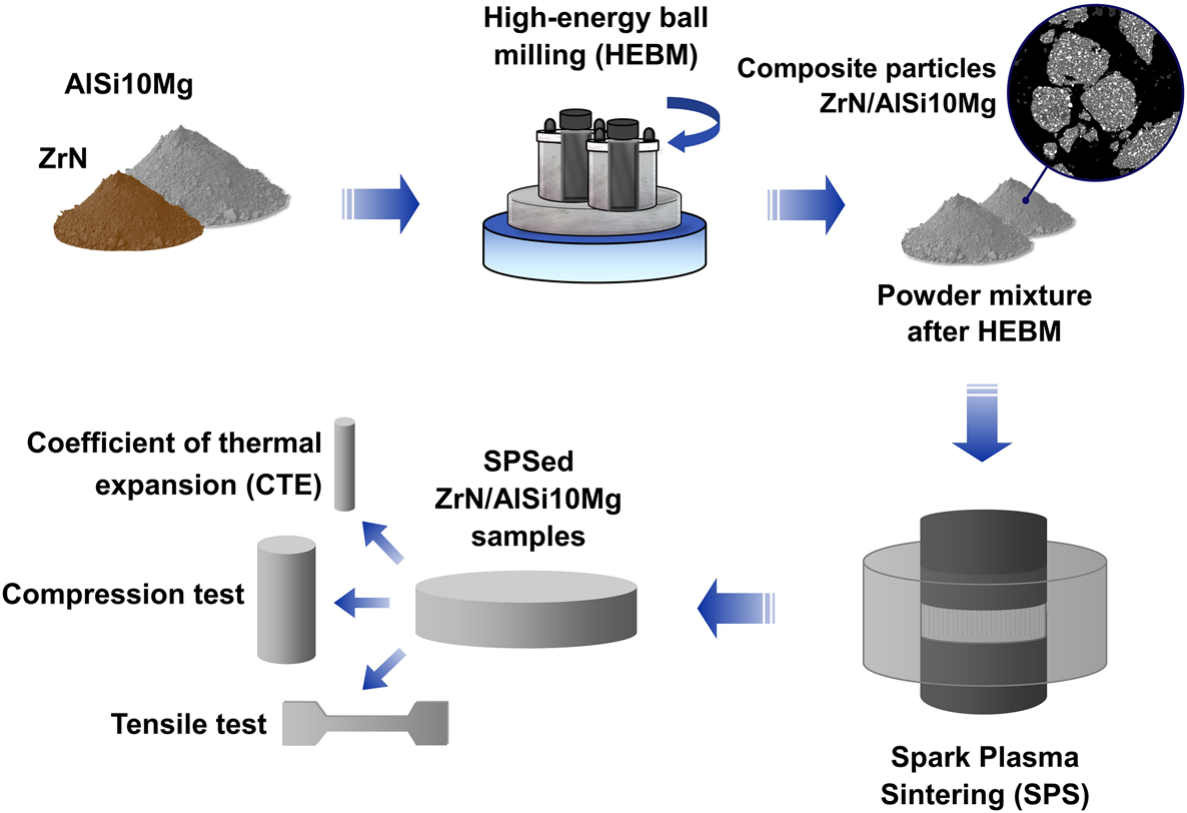
Simplified synthesis scheme.

In this study, the ZrN/AlSi10Mg compact composites were produced by the powder metallurgy method. Figure 1 shows the schematic representation of the fabrication process of the composites. Initially, powder mixtures of AlSi10Mg with 10, 20, and 30 wt% ZrN (sample names: 10Z, 20Z and 30Z respectively) were subjected to high-energy ball milling (HEBM) in an Activator-2S planetary ball mill (Chemical Engineering Plant Ltd., Russia). The HEBM of the mixture was carried out under an argon atmosphere (0.6 MPa) in 250 ml steel jars with 6-mm steel balls as grinding media at a 15:1 ball-to-powder mass ratio (300g:20g). The rotation speeds of the planetary disk and jars were equal and amounted to 650 rpm, HEBM duration of 10 min. To prevent the sticking of powders to the grinding bodies and the walls of the pots, stearin was added.

In order to obtain ZrN/AlSi10Mg compacts, the prepared AlSi10Mg with 10, 20, or 30 wt% ZrN powder mixtures were sintered in a Labox 650 spark plasma sintering unit (SinterLand, Japan) in vacuum under a load of 50 MPa at 500 °C for 20 min. Heating was carried out at a constant rate of 100 °C/min. Initial AlSi10Mg powder with no additives was sintered in the same mode without preliminary HEBM. Through this process, disks with a diameter of 30 mm and a thickness of 7–8 mm were fabricated.

### Characterization and properties

The phase composition of the bulk aluminum-matrix composites ZrN/AlSi10Mg was studied using a DRON X-ray diffractometer (Burevestnik, Russia) with Cu-Kα radiation in the step-by-step scanning mode (shooting step is 0.1°) with an exposure of 2 s. Phases were identified using PDF 2 (2004) database.

The microstructures of the powders and bulk samples after HEBM and SPS were investigated via a JEOL JSM7600F scanning electron microscope (JEOL Ltd., Japan) equipped with an X-MAX 80 mm^2^ attachment (Oxford Instruments, UK) for X-ray microanalysis. Images were obtained at an accelerating voltage of 15 kV. The fine structure was studied by high-resolution transmission electron microscopy (TEM) on a JEM-2100 microscope (Jeol, Japan). The High-Resolution Cross-sectional Transmission Electron Microscopy (x-HRTEM) data were obtained from the above lamella with an electron microscope JEM-2100 Plus (JEOL, Tokyo, Japan) operating at 200 kV. The electron beam current in the scanning transmission electron microscopy (STEM) mode was 1 nA. The lamella was treated with a focused ion beam at an accelerating voltage of 30 kV. During the cross section, the current was 0.79 nA, and during polishing, it was 24 pA. Particle size analysis was performed on a Bettersizer ST analyzer (Bettersize Instruments LTD, China) with wet dispersion in distilled water. The flow rates of the powders of a weighted quantity of 50 g were assessed using a Hall flowmeter installation (ASTM B213-17).

Relative density (ρ) was determined as the ratio of pycnometric density (ρ_p_) measured using an Ultrapycnometer1000 helium pycnometer (Quantachrome Instruments, USA) and theoretical density calculated by rule of mixture.

Vickers hardness was measured using a Durascan-70 digital hardness tester (Struers ApS, Denmark) at a maximum load of 20 N and an exposure time of 10 s. The measurement of Young's modulus and nanohardness was conducted on a Micro-Hardness Tester (CSM Instruments, Switzerland) under an applied load of 50 mN and at an exposure time of 10 s. The Oliver–Pharr technique was used to interpret the collected data^33^.

Tensile testing was carried out on samples with a rectangular cross-section of 2 × 6 mm^2^ and a length of 15 mm using an Instron 5966 universal testing machine (Instron, USA). Compress testing was conducted for cylindrical samples with a height-to-diameter ratio of 2:1 on a hydraulic testing machine PGM-100MG4 (SKB StroyPribor LLC, Russia). The loading rate in tension and compression was 5 and 0.6 MPa/s, respectively. The CTE was studied on cylindrical samples with a diameter of 4 mm and a height of 10 mm on a quenching dilatometer DIL805A/D (TA Instruments, Germany) at a heating rate of 5 °C/min. At least five hardness and tensile/compress strength measurements were taken for each sample.

## Results and discussion

### Characterization of powder mixtures subjected to HEBM

**Figure 2:**
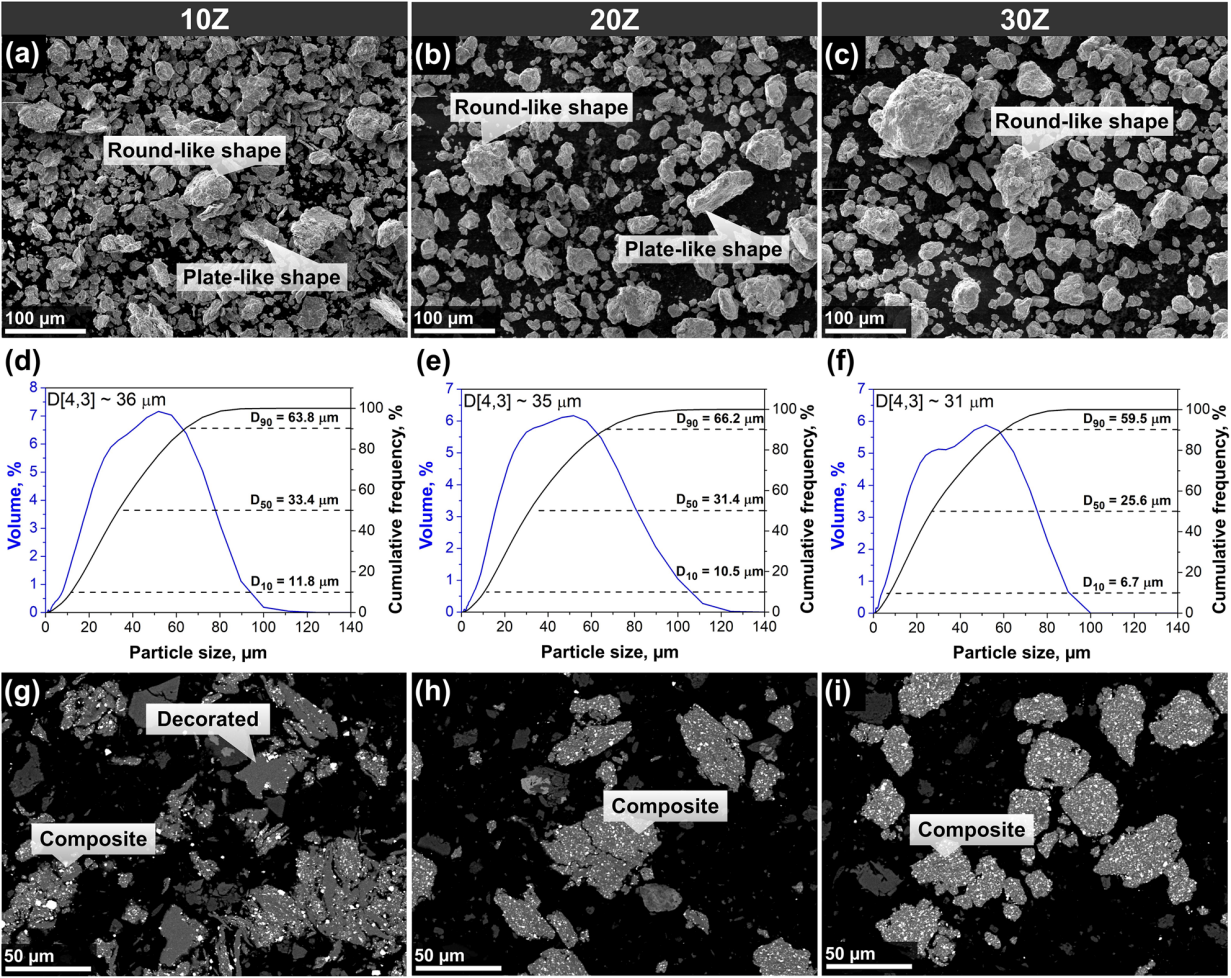
Morphology of ZrN/AlSi10Mg powder composites. (**a**–**c**, **g**–**i**) SEM images and (**d**–**f**) particle size distribution curves of the (**a**, **d**, **g**) 10Z, (**b**, **e**, **h**) 20Z, and (**c**, **f**, **i**) 30Z powders.

In order to form composite particles and, therefore, evenly distribute ZrN in the AlSi10Mg matrix, powder mixtures with 10, 20, and 30 wt% ZrN were subjected to HEBM. The SEM images and particle size distribution curves of resulting powders are demonstrated in Fig. 2. Introduction of 10 wt% ZrN has led to noticeable changes in morphology and has caused a decrease in particle size compared to the original AlSi10Mg powder. In addition to plate-like particles, round-like particles (Fig. 2a) are observed in the composite powders 10Z, which formation, we suppose, has been caused by hardening during the HEBM process. On the other hand, composite powders 20Z (Fig. 2b) consist predominantly of round-like particles; however, plate-like particles can be also observed occasionally. The particles of 30Z powder (Fig. 2c) are more spherical than others containing lower ZrN fraction.

According to the size distribution data, the particle size for 10Z composite varies from 0.5 to 120 μm, and De Brouckère diameter (D[4,3]) equals ~ 36 µm (Fig. 2d). As both 20Z and 30Z compositions are characterized by close D[4,3] values of ~ 35 and ~ 31 µm, respectively (Fig. 2e,f), which is slightly lower compared to the powder 10Z. Despite that the distribution curves of all samples are similar and D[4,3] values are almost identical, the flow rate of the powders significantly decreases with decreasing ZrN content, which is probably connected to morphological differences. The 10Z и 20Z powders do not show flowability, on the contrary, the flow rate of 30Z is found to be 18 s.

XRD patterns of the powder mixtures subjected to HEBM are presented in Fig. S3. In the case of all powder mixtures, there are three well-crystalline phases: α-Al Fm-3m (225), Si Fd-3m (227), and ZrN Fm-3m (225). The lattice parameters of Al, Si, and ZrN are found to be 0.4045, 0.5436, and 0.4567 nm, respectively. The parameters remain constant for all mixtures and are comparable with the feedstock powder, which confirms the absence of chemical interaction between the components during the HEBM process. For mixtures with 10, 20, and 30 wt%, the amount of ZrN phase calculated by the Rietveld method was 9, 19.2, and 29.6 wt%, respectively. Therefore, ZrN losses resulting from powder adhesion to milling media and pot walls during HEBM do not exceed 1 wt% and are considered insignificant.

The particles of a ductile component (AlSi10Mg) plastically deform upon collision with grinding media, which leads to flattening the particles, while the particles of a fragile component (ZrN) are split into smaller ones. As it is known, there is a critical deformation degree upon which cold welding takes place^34^. One should expect that during the HEBM local deformation near ZrN particles increases, which improves the cold-welding process, as it is observed in 6061 aluminum alloy with AlN^35^. This may explain why particles are flattened at a low additive content of 5 wt% of ZrN (Fig. S4), showing similar behavior to other ductile metals where plastic deformation prevails^36–38^. An increase in the number of fragile and hard particles increases the number of areas where severe plastic deformation occurs that, in turn, facilitates the grinding and cold welding of AlSi10Mg. Accordingly, the process of cold welding begins earlier than in systems with lower ZrN concentrations (Fig. S5). Upon this process, grounded ZrN particles are disposed between flattened AlSi10Mg ones, i.e. they are located on the boundaries of AlSi10Mg. Repeatable plastic deformation, grinding, and cold welding cause the formation of round-shaped composite particles. The cross sections of 10Z sample show that there are two particle types: (i) composite particles with ZrN homogeneously distributed in AlSi10Mg matrix; (ii) decorated particles where ZrN is located along the surface of deformed AlSi10Mg particles. Other particles without ZrN shown in Fig. 2g–i are silicon oxide, which is a component of a resin used for metallographic sample preparation (Fig. S6).

Subsequent increasing the mass fraction of ZrN causes more spherical particles to appear. As a result, the composite particles of 30Z sample are more spherical than others, which is responsible for its flowability. In the case of the powder mixtures 20Z and 30Z (Fig. 2h,i, Figs. S7, S8), only composite particles were found. One more explanation for the variation in powder morphologies with low (Fig. S4) and high (Fig. 2c,i) ZrN content is that these ceramic particles can serve as additional milling media^35,37,39^.

Overall, high-energy HEBM processing (650 rpm) in a planetary ball mill for 10 min promotes the formation of ZrN/AlSi10Mg composite particles. Zirconium nitride serves as the milling medium, significantly reducing the duration of plastic deformation of the metal powder and activating the fracture mechanism. This results in the formation of composite particles with a rounded shape within the size range of approximately 31–36 µm and a relatively uniform distribution of reinforcing phase in the metal matrix.

### Spark plasma sintering

The main morphological and chemical transformations in the composites, affecting their functional properties occur during the SPS process. To track the changes and study features of morphology and phase composition of the obtained composites XRD and SEM/EDX analysis were performed.

**Figure 3:**
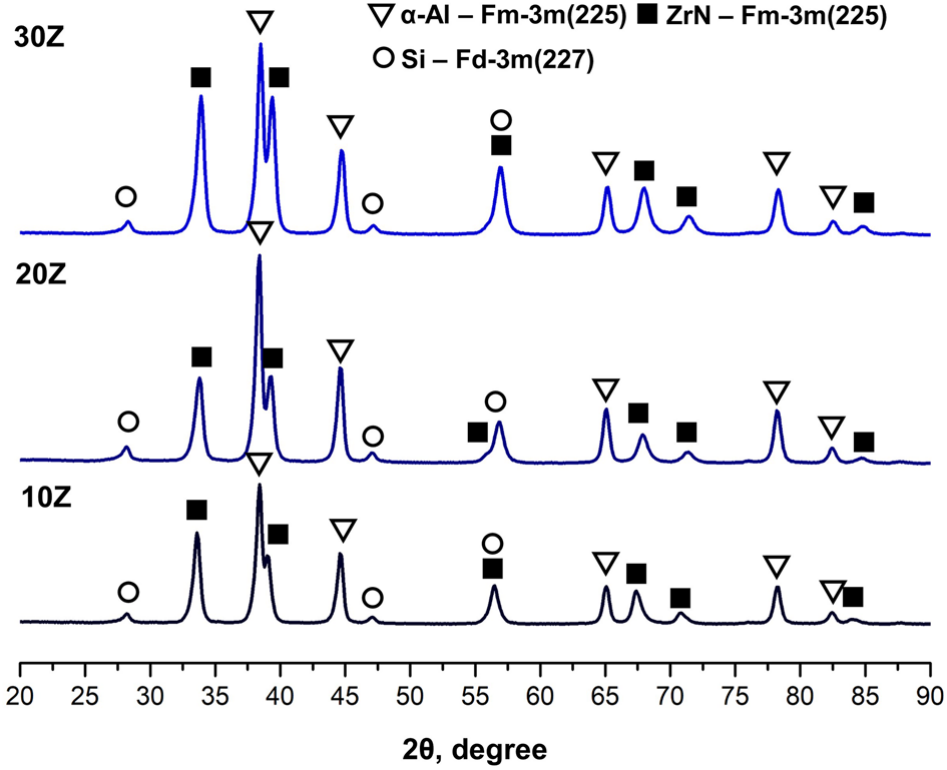
Phase composition of SPS-obtained composites.

Figure 3 shows the XRD profiles of ZrN/AlSi10Mg aluminum-matrix composites with different weight fractions of ZrN after SPS. The peak positions of the α-Al Fm-3m (225), Si Fd-3m (227) and ZrN Fm-3m (225) phases after SPS remain unchanged compared to the powder mixtures after HEBM, which indicates an absence of chemical interaction between components.

**Figure 4:**
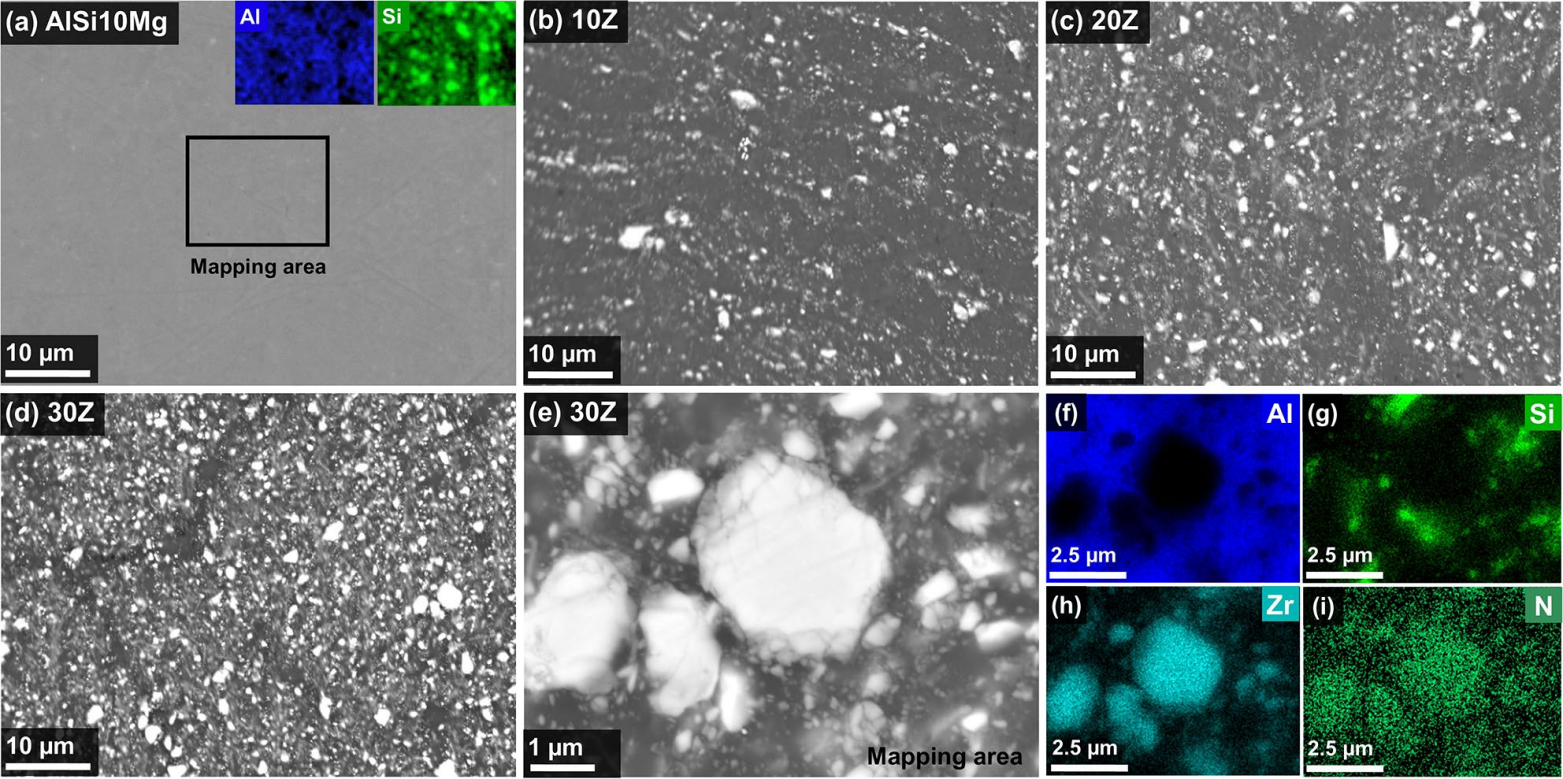
Microstructures and elemental composition of SPS-obtained ZrN/ AlSi10Mg composites. SEM images of (**a**) pristine AlSi10Mg and aluminum matrix composites with (**b**) 10Z, (**c**) 20Z, and (**d**, **e**) 30Z; (**f**–**i**) element distribution maps of Al, Si, Zr, and N in the ZrN/AlSi10Mg composite with 30 wt% ZrN.

The microstructures of sintered AlSi10Mg and composites 10Z, 20Z, 30Z, as well as element distribution maps are shown in Fig. 4. The microstructure of the pure AlSi10Mg (Fig. 4a) has a subtle compositional contrast corresponding to the elements Al and Si, which is in good agreement with the diffraction pattern of AlSi10Mg after SPS (Fig. 3). The microstructures of the ZrN/AlSi10Mg composites (Fig. 4b–e) are characterized by the presence of two types of phases: gray matrix and white inclusion. The analysis of the EDS results (Fig. 4f–i) has revealed that the gray-colored areas correspond to Al and Si, while the white-colored areas—to the ZrN reinforcing particles.

**Figure 5:**
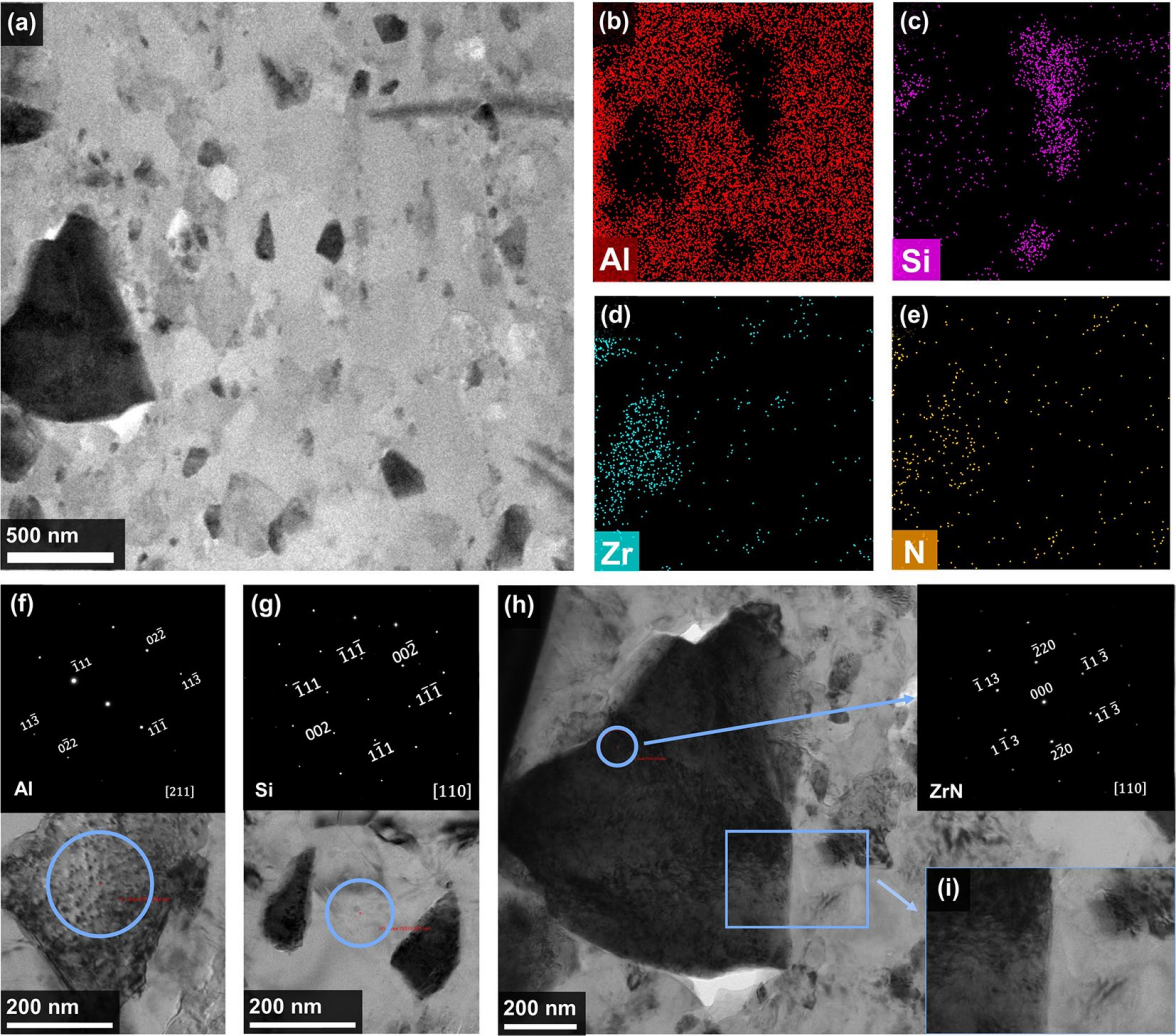
Bright-field STEM images of the 30 wt% ZrN/AlSi10Mg composite. (**a**) TEM-image of the sample and corresponding EDS maps: (**b**) Al, (**c**) Si, (**d**) Zr, (**e**) N; SAED pattern of (**f**) Al, (**g**) Si and (**h**) ZrN particles; (**i**) ZrN and Al phase boundary.

TEM was additionally utilized to accurately examine the phase composition of the 30Z sample (Fig. 5). Three constituent phases were identified in the bright-field mode (Fig. 5a). EDX (Fig. 5b–e) and SAED (Fig. 5f–h) results have revealed that the structure of 30Z composite consists of the matrix containing Al and Si with embedded ZrN particles. The results obtained demonstrate the absence of interaction between ZrN and Al-matrix, and the formation of intermetallics on the phase boundary has not been observed (Fig. 5i).

The density values of the sintered samples determined using the helium pycnometer are 2.6727 ± 0.0005, 3.0833 ± 0.0007, 3.5032 ± 0.0007, 3.9709 ± 0.0003 g/cm^3^ for AlSi10Mg with no additives and with 10, 20, 30 wt% ZrN, respectively. The theoretical densities of the composites were calculated using the rule of mixtures (ρ_AlSi10Mg_ = 2.68 g/cm^3^; ρ_ZrN_ = 7.09 g/cm^3^) and were found to be 3.1144, 3.5208 and 4.003 g/cm^3^ for the 10Z, 20Z, and 30Z, respectively. Therefore, it can be concluded that the relative density of the sintered samples is ≥ 99%.

Overall, the SPS-obtained ZrN/AlSi10Mg composites show no phase or chemical transformation during the sintering process but exhibit significant densification, likely as a result of solid-state mass transfer. The sintering process allows to obtain composites with a nearly fully dense structure.

### Mechanical properties

**Figure 6:**
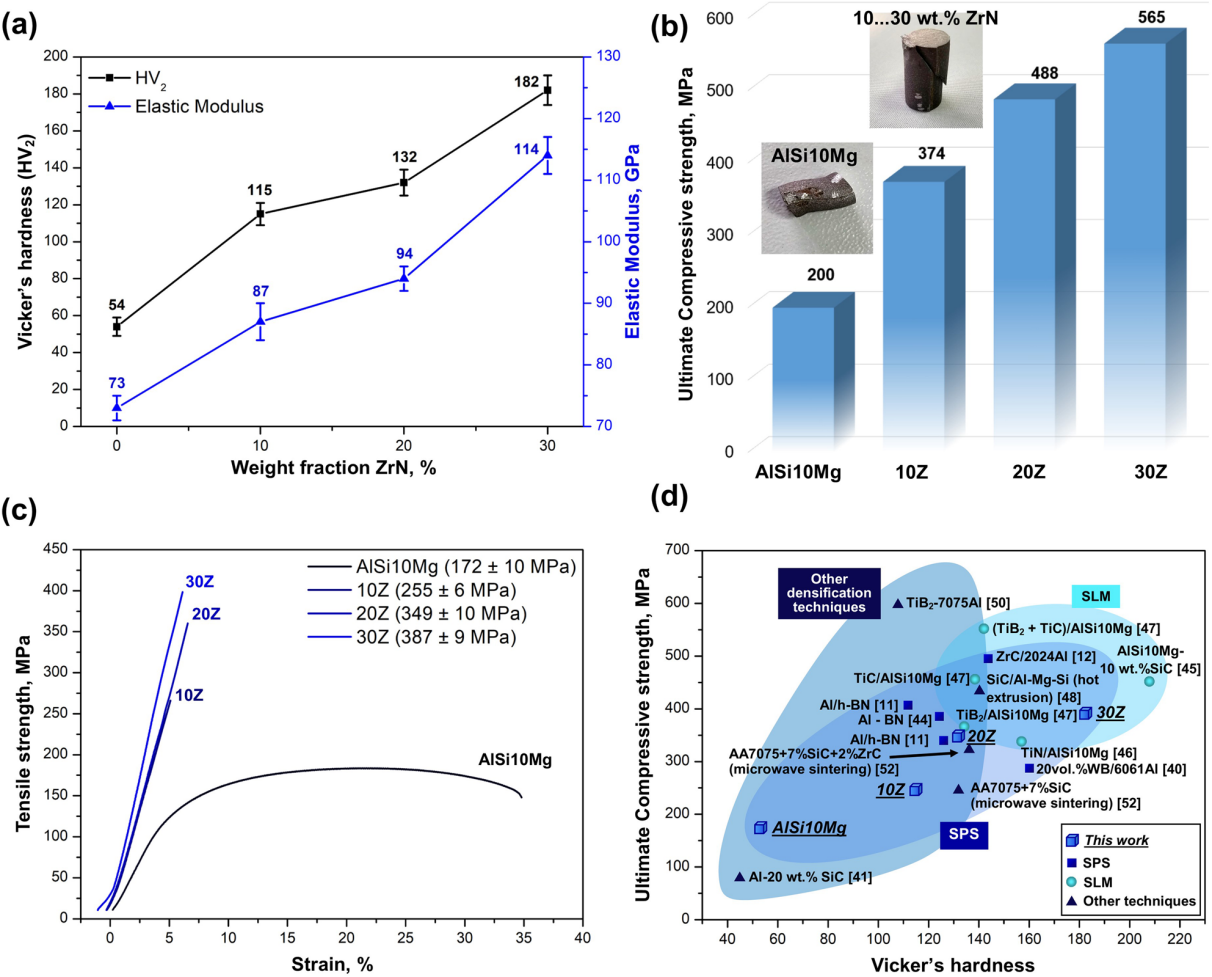
Mechanical properties of the ZrN/AlSi10Mg composites. (**a**) Hardness and elastic modulus, (**b**) ultimate compressive strength, (**c**) tensile curves of the pure AlSi10Mg and ZrN/AlSi10Mg composites with different mass fraction of ZrN, (**d**) ultimate tensile strength and hardness of SPSed ZrN/AlSi10Mg with 10, 20, 30 wt% compared to other aluminum matrix composites.

Mechanical properties (hardness, elastic modulus, compressive, and tensile strength) were measured on the sintered AlSi10Mg and ZrN/AlSi10Mg samples, and the results are shown in Fig. 6.

Figure 6a shows the HV_2_ hardness and elastic modulus depending on the ZrN fraction. The average hardness of the AlSi10Mg aluminum alloy after SPS is 54 ± 5 HV_2_. The hardness of the composites is substantially higher and gradually increases from 115 ± 6 HV_2_ (10Z) to 182 ± 8 HV_2_ (30Z) with the increasing mass fraction of ZrN, which is respectively 112% and 237% higher compared to pure AlSi10Mg. The indentation test has shown that the elastic modulus changes similarly demonstrating a maximum at 114 ± 3 GPa (30Z), which is 56% greater than that of pure AlSi10Mg (73 ± 2 GPa).

The ultimate compressive strength (UCS, Fig. 6b) was measured on cylindrical specimens with a height-to-diameter ratio of 2:1. As can be seen, UCS monotonically increases with the increasing mass fraction of ZrN. The highest UCS value of 565 MPa is observed for the sample 30Z, which is 183% higher compared to pure AlSi10Mg, for which the UCS value is 200 MPa (the value was recorded before necking occurred). Figure 6b shows the appearance of the samples after compression tests. An applied load to the AlSi10Mg sample has caused plastic deformation leading to a change in the cross-sectional area and a displacement of the upper part of the sample relative to the axis along which the load was applied. All the ZrN/AlSi10Mg composites failed by shear banding oriented about 45° with respect to the loading direction.

Figure 6c shows the tensile curves of the AlSi10Mg and ZrN/AlSi10Mg composites. Pure AlSi10Mg deforms significantly, and the elongation is 25%, but a yield plateau has not been observed. On the other hand, the ZrN/AlSi10Mg composites do not show ductility, and the fracture is observed in the elastic region. The introduction of the ZrN ceramic additive has a positive effect on the strength of AlSi10Mg. In the range of 10–30 wt% ZrN tensile strength increases monotonically, reaching a maximum value of 387 ± 9 MPa for the 30Z, which is 125% higher compared to the pure AlSi10Mg. Figure 6d shows the values of Vicker's hardness and ultimate tensile strength of the ZrN/AlSi10Mg (cubes) in comparison with AMCs obtained by various compaction methods.

**Figure 7:**
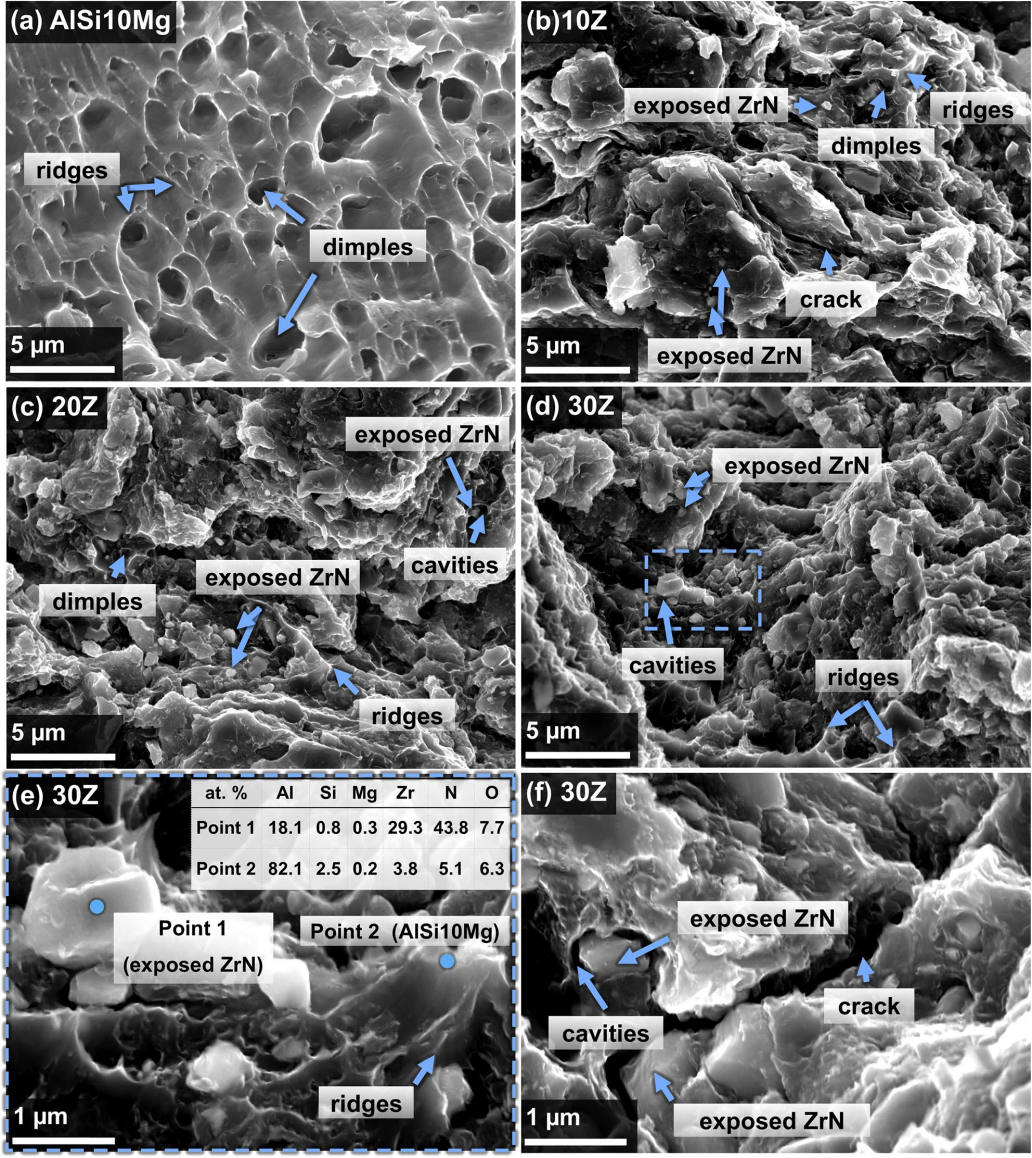
Fracture topography after tensile tests. SEM images of (**a**) pure AlSi10Mg and ZrN/AlSi10Mg composites with (**b**) 10, (**c**) 20, (**d**–**f**) 30 wt% ZrN.

The fracture surfaces of the AlSi10Mg and ZrN/AlSi10Mg samples with different ZrN contents after tensile tests are demonstrated in Fig. 7. The fracture surface of the AlSi10Mg (Fig. 7a) appears to be rough having dimples of different sizes with sharp ridges, which indicates ductile fracture of the material and is in good agreement with the stress–strain curve (Fig. 6c). An increase in the mass fraction of ZrN to 10 wt% (Fig. 7b) leads to a noticeable change in the morphology of the fracture surfaces. The number of dimples has noticeably decreased in comparison with pure AlSi10Mg, and ridges are found in some places on the fracture surface. Additionally, exposed ZrN and cracks are observed. As the mass fraction of ZrN increases to 20 and 30 wt% (Fig. 7c–f), the amount of exposed ZrN increases. At higher magnification, it can be seen (Fig. 7e,f) that some exposed ZrN particles are located in the cavities and are separated from the cavity walls, while the rest are firmly fixed in the matrix. Some exposed ZrN particles show signs of chipping. From SEM images of the fracture surface of the ZrN/AlSi10Mg composites, one can conclude that the fracture is of the mixed nature, in which the brittle fracture mechanism (chips on ZrN particles) prevails over the ductile one (AlSi10Mg dimples and ridges).

**Figure 8:**
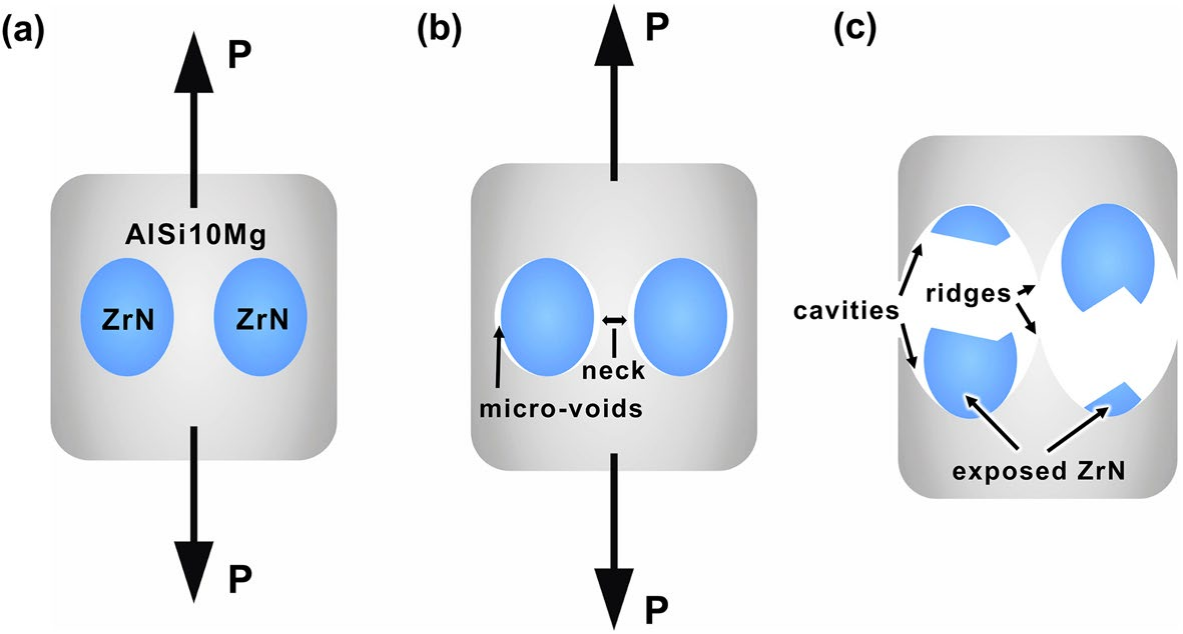
Tensile fracture mechanism of ZrN/AlSi10Mg composites.

Figure 8 shows a schematic representation of the fracture mechanism of the ZrN/AlSi10Mg composites under tension. Initially (Fig. 8a,b), the matrix undergoes plastic deformation under tensile stresses, and the localization of deformation occurs resulting in neck formation (Fig. 8b), while the ZrN particles remain unchanged. Due to the flow of AlSi10Mg and, consequently, a change in the volume of the matrix around the ZrN particles, partial decohesion occurs at the AlSi10Mg-ZrN boundary causing micro-voids formation in the vicinity of the ZrN particles. Further deformation promotes necking development, and the reinforcing ZrN particles begin to experience the load. Simultaneously, the micro-voids nucleated at the AlSi10Mg-ZrN boundary aggregate, and cavities appeared around the ZrN particles. Eventually, the necks tear apart leaving ridges responsible for the fracture surface morphology. The resulting cracks propagate along the AlSi10Mg-ZrN boundary, and the applied load is fully transferred to the ZrN particles, which are firmly bonded to the AlSi10Mg matrix. HEBM promotes the formation of micro-cracks in ZrN particles. The stress concentration at the tips of these defects that arises under the influence of the load leads to the destruction of ZrN particles by chipping. As a result, the composites are destroyed (Fig. 8c). A similar failure mechanism has been observed in other AMCs with ceramic reinforcements^40–43^.

The mechanical properties of the ZrN/AlSi10Mg and some AMCs are summarized in Table 1. The ZrN/AlSi10Mg composites obtained exhibit mechanical properties comparable to other AMCs obtained by various compaction methods (Fig. 6d, squares—SPS^11,12,40,44^, circles—SLM^45–47^, triangles—other methods, including microwave sintering, stir casting process, etc.^48–52^). The composite 30Z has higher ultimate tensile strength compared to 53% of AMCs represented in Fig. 6d, and only one AMC exceeds the ZrN/AlSi10Mg in terms of hardness. For AlSi10Mg-10SiC^45^, obtained by the SLM method, the HV_0.1_ value is 208.5, and for the ZrN/AlSi10Mg the hardness value is slightly lower HV_2_ = 182, but this difference is primarily due to the difference in the applied loads.

**Table 1:** Mechanical properties of the AlSi10Mg, ZrN/AlSi10Mg, and other aluminum-matrix composites.

Sample	Densification technique	Hardness	UCS, MPa	UTS, MPa	Strain, %	References
ZrC/2024Al	SPS T6 heat treatment	143.7 ± 2 HV_0.02_	–	495 ± 4	12.0 ± 0.1	^12^
10 wt% SiC/AlSi10Mg	SLM	208.5 HV_0.1_	–	450	–	^45^
TiN/AlSi10Mg	SLM	105.9 ± 2.1 HV_0.1_	–	336.8 ± 1.5		^46^
(ZrAl_3_ + AlN)/Al	Pressureless sintering	71.7 ± 2.1 HBW	–	272.8 ± 12.6	10.7 ± 2.6	^49^
TiB_2_/7075 Al	Torsion extrusion	10.75 HV_20_	–	608	11.4	^50^
(SiC + ZrC)/AA7075	Microwave sintering	136 HV_0.2_	389	321	17.5	^52^
AlSi10Mg	SPS	54 ± 5 HV_2_	200 ± 11	172 ± 10	23 ± 2	This work
10Z	SPS	115 ± 6 HV_2_	374 ± 13	255 ± 6	–	This work
20Z	SPS	132 ± 6 HV_2_	488 ± 10	349 ± 10	–	This work
30Z	SPS	182 ± 8 HV_2_	565 ± 15	387 ± 9	–	This work

Mechanical properties significantly depend on the fabrication technology and matrix materials used. Therefore, it is more correct to evaluate the effectiveness of reinforcing additives compared with the original metal or alloy with no additives. In this work, the addition of 10–30 wt% ZrN to the AlSi10Mg alloy has increased hardness, compressive, and tensile strength by 112–237%, 87–183%, and 48–125%, respectively. The high mechanical properties of the ZrN/AlSi10Mg composites may be due to both strain hardening during the HEBM process and the reinforcement effect. As previously said, during the HEBM process, the aluminum matrix undergoes plastic deformation resulting in decreasing grain size eventually leading to strain hardening of the composites^53,54^. Moreover, reinforcement may also contribute to an increase in mechanical properties due to the following: (1) transferring the applied load from the plastic AlSi10Mg matrix to solid ZrN particles; (2) grain refinement due to the addition of ceramic ZrN particles resulting in enhancing properties according to the Hall–Petch relation; (3) elastic stress appearing because of a severe difference between the CTE of AlSi10Mg (20.5 × 10^−6^ °C^−1^) and ZrN (7.24 × 10^−6^ °C^−1^^23^).

Overall, the results highlight that the synergistic effects of deformation strengthening during HEBM and reinforcement lead to a significant enhancement in the mechanical properties of ZrN/AlSi10Mg. This improvement becomes more pronounced with an increasing mass fraction of ZrN. However, during the tensile testing of the composites, produced via HEBM followed by SPS, a mixed fracture mode is evident, where the brittle fracture mechanism prevails over the ductile one.

### Coefficient of thermal expansion

**Figure 9:**
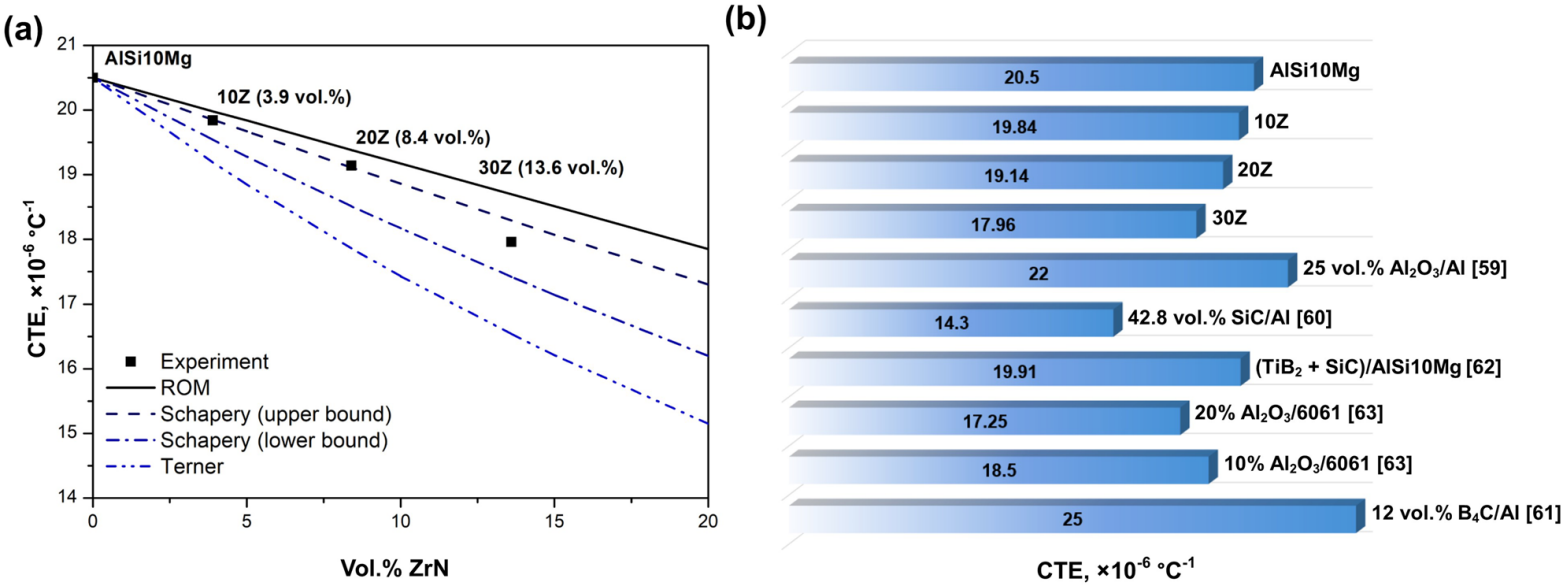
CTE values of the ZrN/AlSi10Mg composites. (**a**) Experimentally measured CTE of the AlSi10Mg and ZrN/AlSi10Mg with different ZrN content, as well as calculated CTE in accordance with the ROM and Schapery models; (**b**) CTE of the ZrN/AlSi10Mg obtained and other AMCs.

Figure 9a shows the dependence of the experimentally measured CTE of ZrN/AlSi10Mg on the volume fraction of ZrN, as well as the calculated CTE by the ROM (rule of mixture) models^55^, Turner^56^, and Schapery^57^. The experimentally measured CTE of the AlSi10Mg and ZrN/AlSi10Mg composites are indicated by solid squares, and the results of theoretical calculations are presented as lines: continuous (ROM), dash double dot (Turner), dashed (Schapery upper bound), dash-dot (Schapery lower bound). Detailed descriptions of the theoretical calculations of the CTE are presented in the Supplementary file.

The CTE values obtained experimentally have the greatest agreement with the Schapery model (Fig. 9a). CTE of the composites 10Z and 20Z lies on the dotted line corresponding to Schapery’s upper limit. At the same time, the CTE of the composite 30Z is located slightly below Schapery’s upper limit. The CTE values calculated by the Turner model and ROM do not match the experimental data (Table 2) and do not allow the prediction of CTE with sufficient accuracy. The good convergence of the experimental CTEs with Schapery’s model may indicate a good interconnection between the components of the AlSi10Mg and ZrN composite^58^, as well as the presence of a complex mechanical interaction between them. Therefore, Schapery’s model, which considers shear and volumetric stresses, is the most suitable for predicting the CTE of ZrN/AlSi10Mg.

**Table 2:** Thermal expansion coefficients of ZrN/AlSi10Mg and other AMCs.

Sample	α_c_, × 10^−6^ °C^−1^					
	Experiment	ROM	Terner	Schapery (upper bound)	Schapery (lower bound)	References
42.8 vol% SiC/Al	14.3	–	–	–	–	^60^
12 vol% B_4_C/Al	~ 25	~ 24	~ 19	~ 23	~ 21	^61^
(TiB_2_ + SiC)/AlSi10Mg	19.91	–	–	–	–	^62,62^
20% Al_2_O_3_/6061	17.25	–	–	16.75	–	^63,63^
AlSi10Mg	20.5	–	–	–	–	This work
10Z	19.8	19.98	19.19	19.85	19.54	This work
20Z	19.1	19.38	17.86	19.11	18.51	This work
30Z	18	18.7	16.54	18.29	17.42	This work

For the 10Z, 20Z and 30Z CTE are 19.8 × 10^−6^ °C^−1^, 19.1 × 10^−6^ °C^−1^, and 18 × 10^−6^ °C^−1^, respectively (Table 2), which is by ~ 3, 7, and 12% lower than for pure AlSi10Mg (20.5 × 10^−6^ °C^−1^). As can be seen from Fig. 9b and Table 2, the ZrN/AlSi10Mg have CTE values comparable to other AMCs^59–63^. For example, the ZrN/AlSi10Mg with 30 wt% ZrN exhibits lower CTE compared to Al-12 vol% B_4_C^61^, AlSi10Mg-SiC-TiB_2_^62^, and 10% Al_2_O_3_/6061^63^. However, the CTE of composites containing SiC^60^ is slightly lower than that of the ZrN/AlSi10Mg.

## Conclusion

In this study, bulk ZrN/AlSi10Mg composites with 10, 20, and 30 wt% ZrN were successfully synthesized. Utilizing suitable HEBM processing parameters, bulk samples with excellent mechanical strengths and thermal properties were fabricated via SPS. The obtained results enable us to conclude the following:

1. With increasing ZrN fraction from 10 to 30 wt%, parameters D50 and D[4,3], corresponding to mean particle size, decrease. At the ZrN content of 10 wt%, ductile AlSi10Mg particles undergo plastic deformation leading to both flattened and spherical morphologies with the formation of composite and round-like particles. As ZrN content increases from 20 to 30 wt%, fragmentation and flattening effects cause the formation of mainly composite round-like particles, while 30 wt% of ZrN provides flowability suitable for additive manufacturing.
2. XRD, SEM, and TEM observations have revealed that bulk ZrN/AlSi10Mg composites sintered by SPS at 500 °C do not demonstrate any phase and chemical transformations under sintering. The cell parameters and peak positions of main phases remain unchanged upon SPS compared to the powder mixtures after HEBM.
3. The incorporation of ZrN particles significantly enhanced the mechanical properties of bulk ZrN/AlSi10Mg composites, resulting in a remarkable increase in hardness, elastic modulus, compressive strength, and tensile strength. These mechanical properties exhibited a consistent improvement with an increase in ZrN content from 10 to 30 wt. For a composite with 30 wt% ZrN, the hardness, elastic modulus, compressive strength, and tensile strength values were 182 ± 8 HV2, 114 ± 3 GPa, 565 ± 15 GPa, and 387 ± 9 GPa, respectively. These values represent a substantial improvement of 237%, 56%, 183%, and 125% compared to the non-strengthened AlSi10Mg alloy.
4. Under tension, the ZrN/AlSi10Mg composites exhibit a complex fracture mechanism. The mechanism involves plastic deformation in the matrix, which leads to neck formation. Partial decohesion at the AlSi10Mg-ZrN boundary causes micro-voids, and subsequent deformation results in the tearing of necks, leaving ridges on the fracture surface. The cracks propagate along the boundary, transferring the load to ZrN particles bonded to the matrix. Moreover, HEBM contributes to micro-crack formation in ZrN particles, ultimately leading to composite destruction.
5. The experimentally determined coefficients of thermal expansion (CTEs) for the ZrN/AlSi10Mg composites align well with Schapery's model, considering both volumetric and shear stresses. The CTE values for ZrN/AlSi10Mg composites with 10%, 20%, and 30% ZrN content are 19.8 × 10^−6^ °C^−1^, 19.1 × 10^−6^ °C^−1^, and 18 × 10^−6^ °C^−^1, respectively.

The results contribute to the understanding of the synthesis, mechanical behavior, and thermal properties of novel bulk ZrN/AlSi10Mg composites. By changing the amount of additive, the target parameters of the composite can be adjusted: cost, relative density, mechanical, and thermophysical properties. Each compound developed has potential for advanced engineering applications, especially round-like composites, which may be suitable for additive manufacturing.

### Supplementary Information

Supplementary Information.

## Data Availability

The data from this study are available from the corresponding author upon reasonable request.
