# Supplementary material for: Enhanced microstructure and mechanical properties of ZrN-reinforced AlSi10Mg aluminum matrix composite
Source: Sci Rep. 2024 May 2;14:10152. doi: 10.1038/s41598-024-58614-6 (PMC11066127; doi:10.1038/s41598-024-58614-6)
Supplement: Supplementary file 1 — Supplementary Information. [file 41598_2024_58614_MOESM1_ESM.docx]

**SUPPLEMENTARY INFORMATION**

**Enhanced microstructure and mechanical properties of ZrN-reinforced AlSi10Mg aluminum matrix composite**

Veronika. Suvorova^a^*, Sergey Volodko^a^, Dmitrii Suvorov^a^, Stanislav Chernyshikhin^a^, Andrey Nepapushev^a^, Artem Korol^a^, Lidiya Volkova^b^, Pavel Sokolov^a^, Alexander Khort^c^* and Dmitry Moskovskikh^a^*

*^a^ University of Science and Technology MISIS, Moscow, Russia*

*^b^Institute of Nanotechnology of Microelectronics of the Russian Academy of Sciences Moscow, Russia*

^c^ *KTH Royal Institute of Technology, Stockholm, Sweden*

**Corresponding authors, e-mail:* [buynevich.vs@misis.ru](mailto:buynevich.vs@misis.ru) (VS), khort@kth.se (AKh), mos@misis.ru (DM)

**Chapter: « Materials and methods»**

**Subsection: «Feedstock powders»**

**
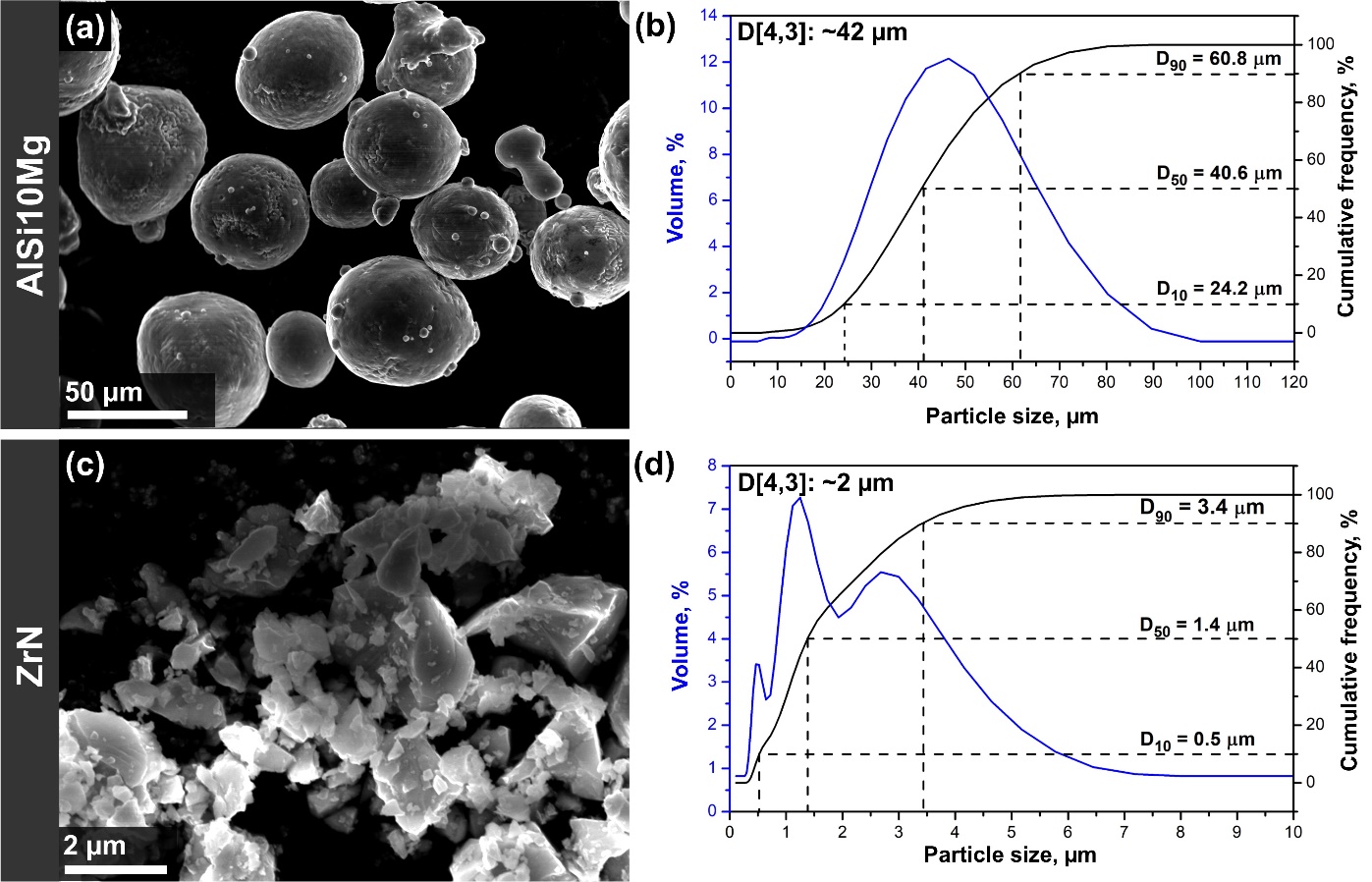
**

**Figure S1. SEM-images of raw powders.** (**a,c**) Morphology and (**b,d**) particle size distribution curves of the feedstock AlSi10Mg and ZrN powders.

**Table S1**. Content of elements in AlSi10Mg alloy

| Alloy | Content of elements, wt.% | | | | | | | | | | |
| --- | --- | --- | --- | --- | --- | --- | --- | --- | --- | --- | --- |
|  | Al | Si | Mg | Fe | Mn | Ti | Zn | Ni | Cu | Sn | Pb |
| AlSi10Mg | 81.7 | 11 | 0.45 | 0.55 | 0.45 | 0.15 | 0.1 | 0.05 | 0.05 | 0.05 | 0.05 |

**
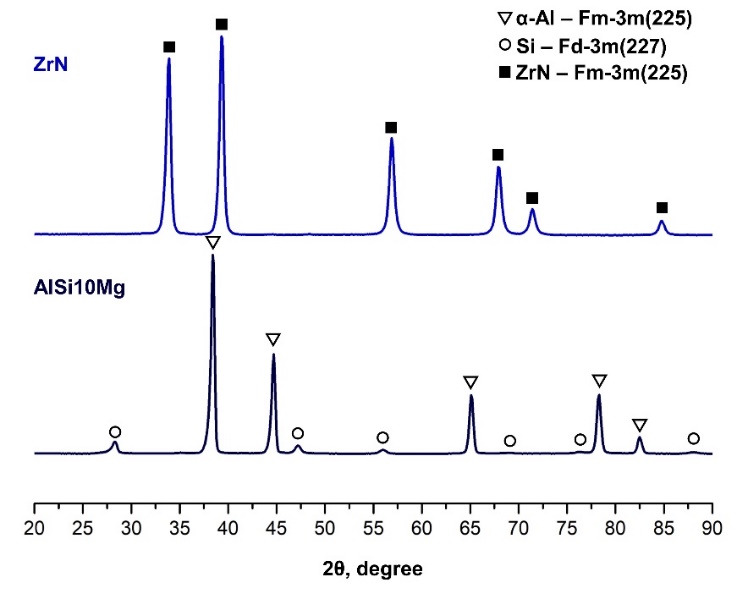
Figure S2.** Diffraction patterns of the AlSi10Mg and ZrN powders.

**Chapter: «Results and discussion»**

**Subsection: «Characterization of powder mixtures subjected to HEBM»**


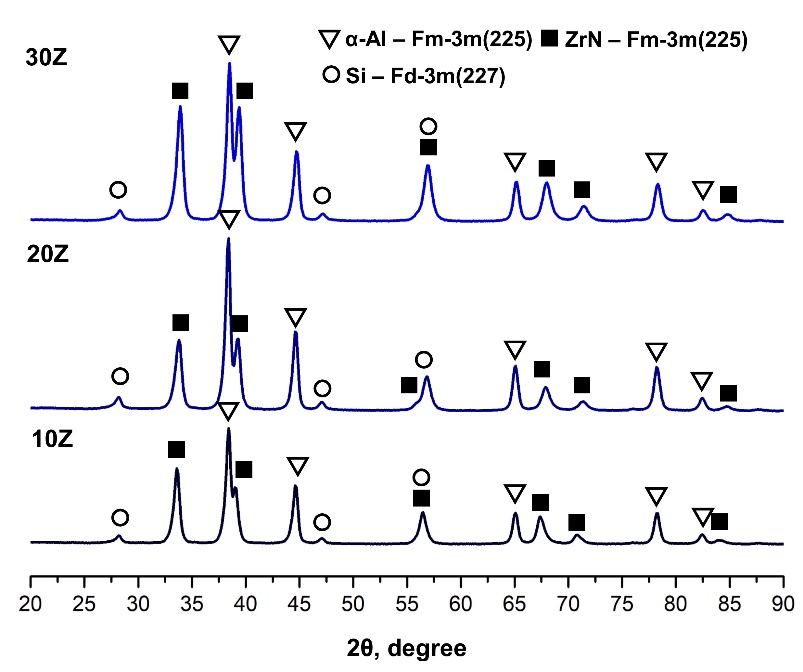


**Figure S3.** Phase composition of HEBM-obtained composites.

**
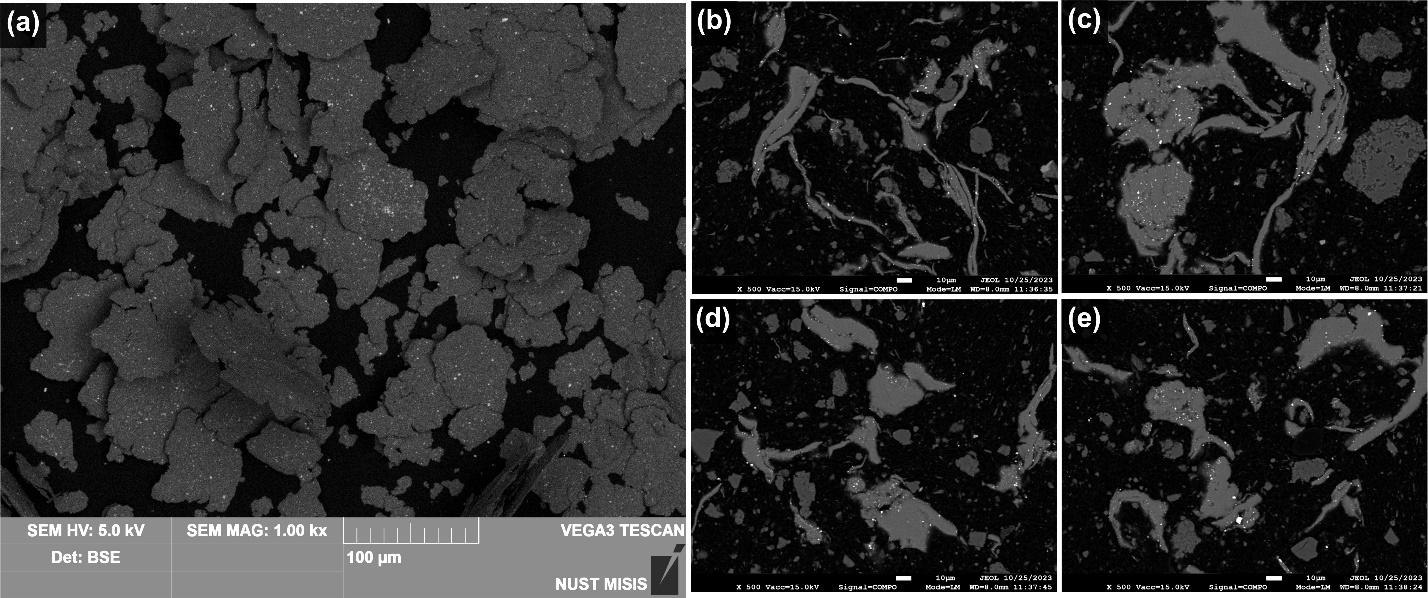
**

**Figure S4. SEM-images of ZrN/AlSi10Mg powder composite with 5wt.% ZrN.**

**(a)** Morphology and **(b-e)** cross section.

**
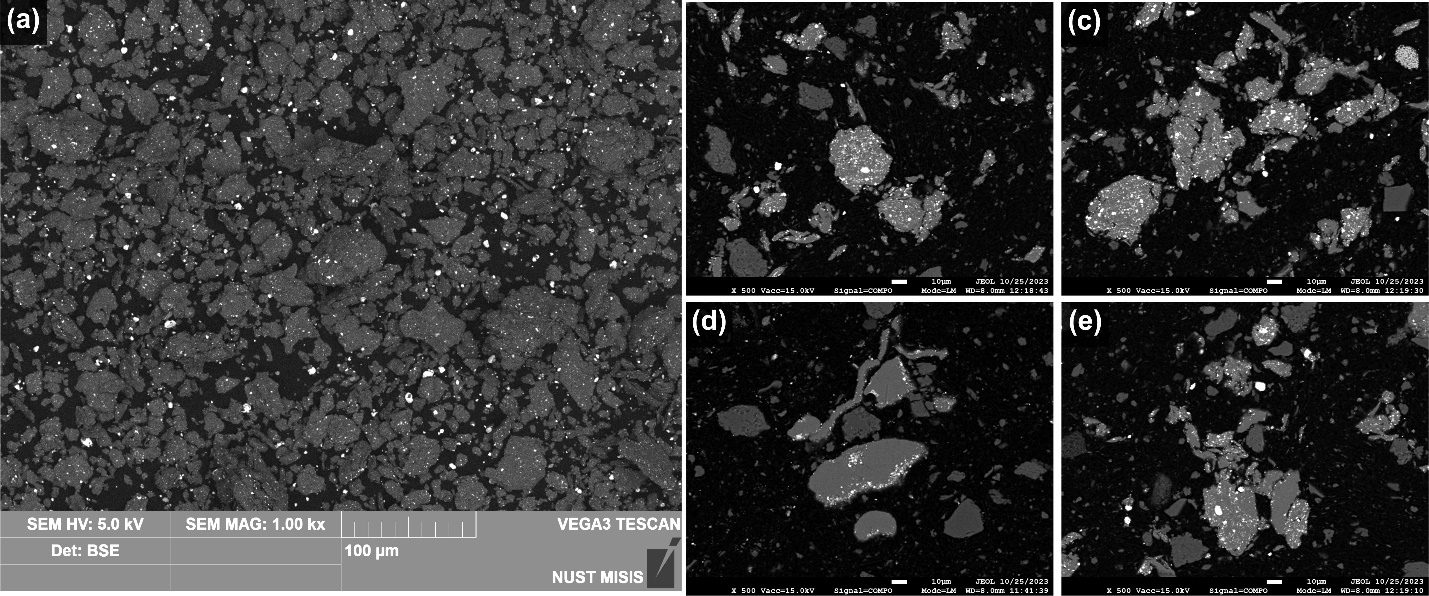
**

**Figure S5. SEM-images of ZrN/AlSi10Mg powder composite with 10wt.% ZrN.**

**(a)** Morphology and **(b)** cross section.

**
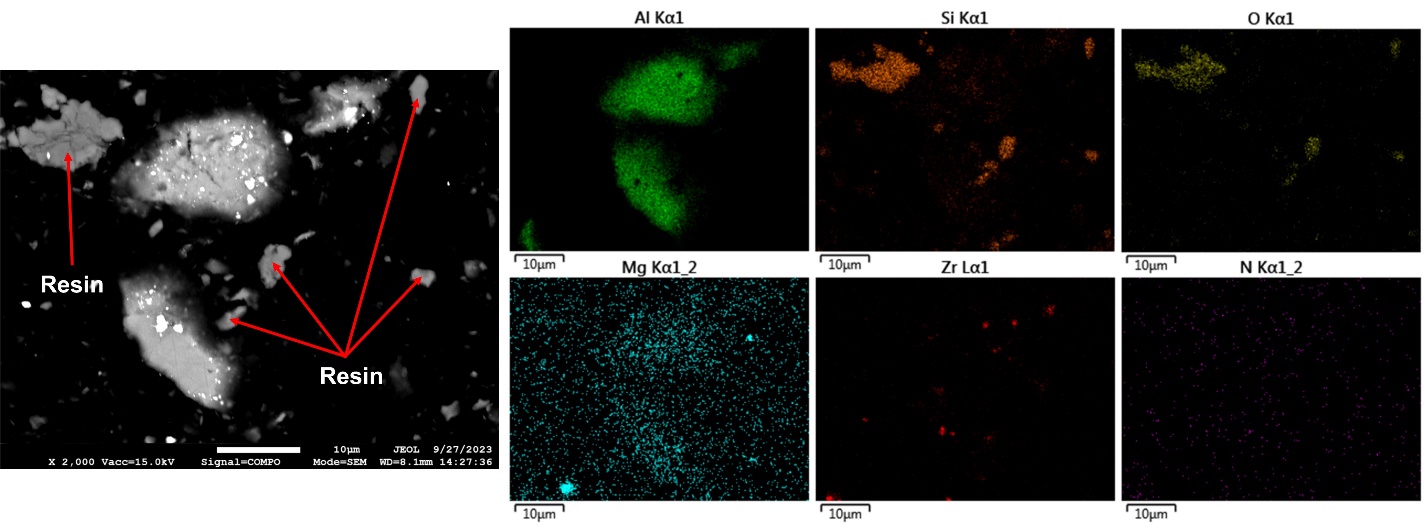
**

**Figure S6. Microstructures and elemental composition** of ZrN/AlSi10Mg powder composite with 10wt.% ZrN.

**
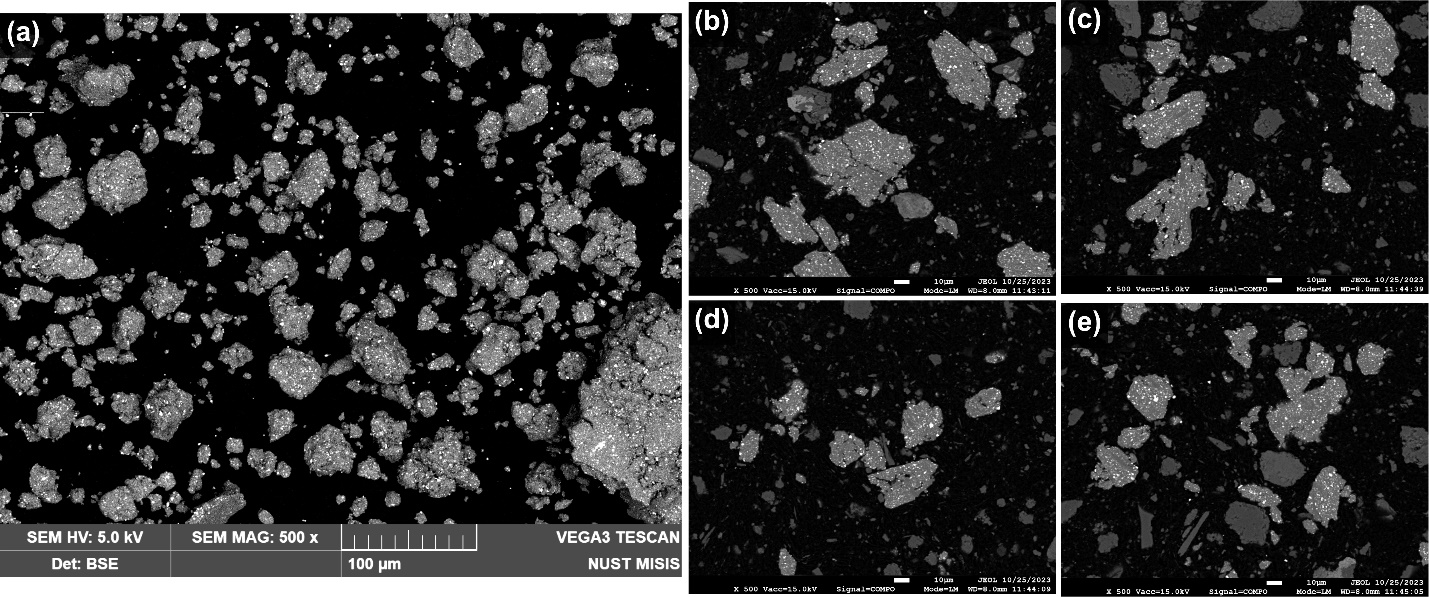
Figure S7. SEM-images of ZrN/AlSi10Mg powder composite with 20wt.% ZrN.**

**(a)** Morphology and **(b)** cross section.

**
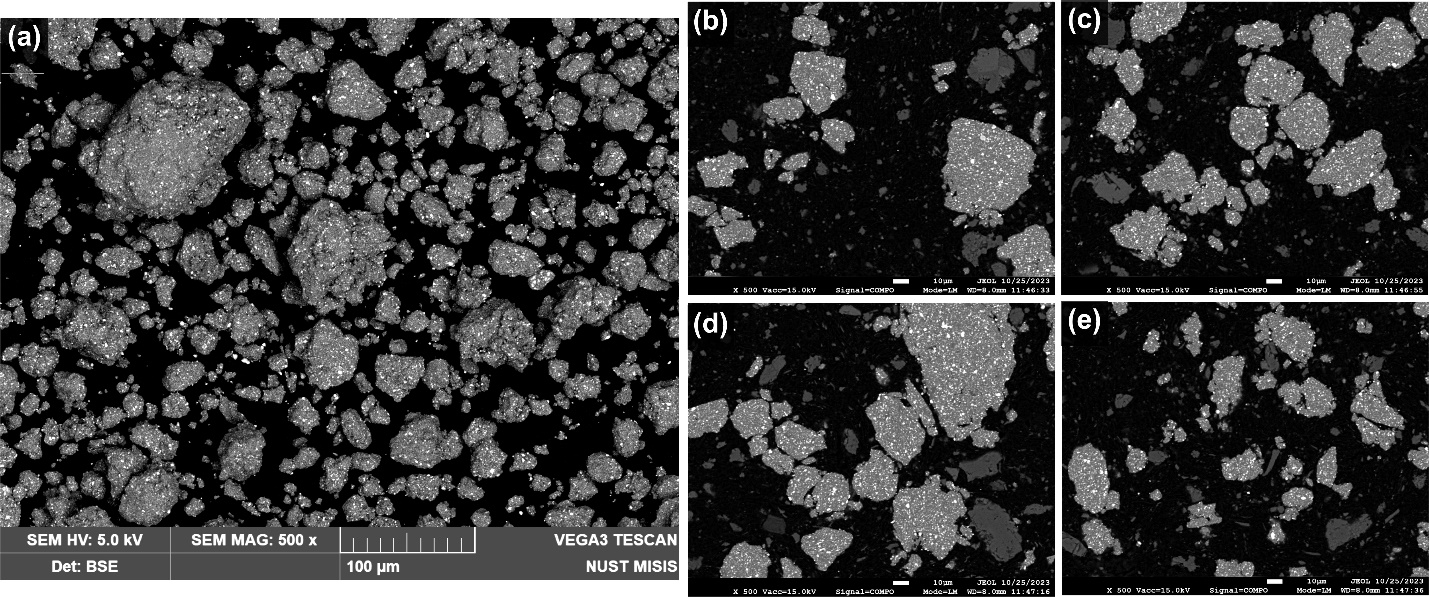
**

**Figure S8. SEM-images of ZrN/AlSi10Mg powder composite with 30wt.% ZrN.**

**(a)** Morphology and **(b)** cross section.

**Subsection: «Coefficient of thermal expansion»**

CTE was calculated using the following models:

The ROM model considers only the CTE of the composite phases and their volume fractions:

$\alpha_{с}= \alpha_{m}V_{m}+ \alpha_{d}V_{d}$ (1)

where α_m_ and α_d_ are CTE of a matrix (AlSi10Mg: 20.1 × 10^-6^ °С^-1^) and reinforcing additives (ZrN: 7.24 × 10^-6^ °С^-1^ [1]); V_m_ and V_d_ – volume fractions.

Turner's model takes the mechanical interaction between the matrix and the reinforcing additives into account, but assumes that both phases experience only volumetric stress:

$\alpha_{c}= \frac{\alpha_{m}K_{m}V_{m}+\alpha_{d}K_{d}V_{d}}{K_{m}V_{m}+K_{d}V_{d}}$ (2)

where K_m_ and K_d_ are bulk modulus of a matrix (3.63 × 10^10^ Pa) and reinforcing additives (1.92 × 10^11^ Pa).

Schapery [2], based on thermoelasticity, suggested a more complex model for evaluation of upper (α_u_, Eq. 4) and lower (α_l_, Eq. 5) boundaries for CTE isotropic composites, which considers not only volumetric stresses, but also shear stresses.

$\alpha_{u}= \alpha_{m}+\frac{K_{d}\left( K_{m}-K_{c}^{l} \right)(\alpha_{d}-\alpha_{m})}{K_{c}^{l}(K_{m}-K_{d})}$ (3)

$\alpha_{l}= \alpha_{m}+\frac{K_{d}\left( K_{m}-K_{c}^{u} \right)(\alpha_{d}-\alpha_{m})}{K_{c}^{u}(K_{m}-K_{d})}$ (4)

where $K_{c}^{u}$ and $K_{c}^{l}$ are upper and lower boundaries of bulk modulus, which are calculated by Hashin-Shtrikman model [3]:

$K_{c}^{u}=K_{d}+\frac{V_{m}}{\left( \frac{1}{K_{m}-K_{d}} \right)+\left( \frac{3V_{d}}{3K_{d}+4G_{d}} \right)}$ (5)

$K_{c}^{l}=K_{m}+\frac{V_{d}}{\left( \frac{1}{K_{d}-K_{m}} \right)+\left( \frac{3V_{m}}{3K_{m}+4G_{m}} \right)}$ (6)

Bulk moduli (K_m_ и K_d_) and shear moduli (G_m и_ G_d_) for AlSi10Mg and ZrN are calculated as:

$K= \frac{E}{3(1-2\nu)}$ (7)

$G= \frac{E}{2(1+\nu)}$ (8)

where E – elastic modulus (73 GPa for AlSi10Mg, 392 GPa for ZrN [4]), ν – Poisson's ratio (0.33 for Al [5], 0.16 for ZrN [4]).

References:

[1] Chen C-S, Liu C-P, Tsao C-YA, Yang H-G. Study of mechanical properties of PVD ZrN films, deposited under positive and negative substrate bias conditions. Scr Mater 2004;51:715–9. https://doi.org/10.1016/j.scriptamat.2004.06.005.

[2] Schapery RA. Thermal Expansion Coefficients of Composite Materials Based on Energy Principles. J Compos Mater 1968;2:380–404. https://doi.org/10.1177/002199836800200308.

[3] Zare Y, Rhee KY. Development of Hashin-Shtrikman model to determine the roles and properties of interphases in clay/CaCO3/PP ternary nanocomposite. Appl Clay Sci 2017;137:176–82. https://doi.org/10.1016/j.clay.2016.12.033.

[4] Christensen AN, Dietrich OW, Kress W, Teuchert WD. Phonon anomalies in transition-metal nitrides: ZrN. Phys Rev B 1979;19:5699–703. https://doi.org/10.1103/PhysRevB.19.5699.

[5] Liao T, Wang J, Zhou Y. Atomistic deformation modes and intrinsic brittleness of Al 4 Si C 4 : A first-principles investigation: A first-principles investigation. Phys Rev B 2006;74:174112. https://doi.org/10.1103/PhysRevB.74.174112.
